# Viral metagenomics combined with non-targeted serum metabolomics reveals the role of enteroviruses in a mouse model of coronary heart disease

**DOI:** 10.1186/s12985-024-02412-z

**Published:** 2024-07-30

**Authors:** Xiang Li, Sihong Liu, Haonan Wu, Bing Li, Yingying Li, Ruoqi Li, Danli Tang, Huamin Zhang

**Affiliations:** 1 Institute of Basic Theory of Traditional Chinese Medicine, China Academy of Chinese Medical Sciences, Beijing, China; 2 Institute of Traditional Chinese Medicine Information, China Academy of Chinese Medical Sciences, Beijing, China; 3 Medical Experimental Research Center, China Academy of Chinese Medical Sciences, Beijing, China; 4 Institute of Chinese Materia Medica, China Academy of Chinese Medical Sciences, Beijing, China

**Keywords:** Enterovirus group, Serum metabolome, Coronary heart disease, Lipid metabolism, *Tsarbombavirus*

## Abstract

**Background:**

Coronary heart disease (CHD) is a common cardiovascular disease that is associated with altered gut microbiota. Enteroviruses, an essential component of the gut microbiome, may play an important role in disease progression. However, the relationship between enteroviruses and CHD remains unclear. The development of high-throughput sequencing technologies has facilitated research on the interconnections between viruses and disease-related metabolites.

**Methods and results:**

Mice were fed a high-fat diet (CHD group) or chow diet (Sham group) for 12 weeks, and ligation of the left anterior descending coronary artery was performed at the end of week 8. After 4 weeks, all animals were euthanised. Subsequently, the animals were evaluated for basic haemato-biochemical parameters and cardiac function, and aorta staining was performed. Based on enteroviral metagenomics and serum UPLC–MS/MS metabolomics analyses, we evaluated the association between enteroviral groups and serum metabolites of CHD mouse model. A high-fat diet and coronary ligation enabled the establishment of the CHD mouse model. Notably, the enterovirus spectrum of the sham group was significantly different from that of the CHD group, with 24 viral communities of different family and species classification, such as *Tsarbombavirus*, *Mingyongvirus*, *Claudivirus*, and *Firehammervirus*, exhibiting significant differences. In addition, 731 Differential metabolites were detected in the serum of both groups of mice. Correlation network analysis revealed a close relationship between various metabolites related to lipid metabolism and different viruses, including *Tsarbombavirus*, *Mingyongviru*s, *Claudivirus*, and *Firehammervirus*.

**Conclusions:**

An animal model of CHD, characterised by lipid disturbance and myocardial ischaemia, was established using a high-fat diet and ligation of the left anterior descending branch of the coronary artery. *Tsarbombavirus*, *Firehammervirus*, *Mingyongvirus*, and *Claudivirus* were associated with metabolites in the lipid metabolism pathway. The results indicate that *Tsarbombaviru*s may be the main genus interacting with CHD-related metabolites in mice. Conclusively, the findings of our study provide novel insights into the potential relationship enterovirus groups and metabolites associated with CHD.

## Background

Cardiovascular disease is one of the leading causes of morbidity and mortality worldwide [1]. It has been predicted that 23.6 million people will die from cardiovascular disease, mainly heart disease and stroke, by 2030. In the United States, 43.9% of the population is expected to develop cardiovascular disease by 2030 [2]. A high-fat diet, smoking, alcohol abuse, and lack of physical activity are all risk factors for coronary heart disease (CHD) [3]. Atherosclerosis, which results from either vascular endothelial damage or foam cell formation, is regarded as the most common pathogenic mechanism underlying CHD; however, the exact mechanisms remain unclear [4, 5].

The gut microbiome includes microorganisms, such as viruses, bacteria, and fungi. With the development of high-throughput technologies, plausible relationship between the gut microbiota and CHD has been extensively investigated. The gut microbiota can change the pathophysiological state of host atherosclerosis by regulating their own ecological balance, causing changes in host metabolism and other aspects that affect inflammation and lipid metabolism, thereby playing an important role in CHD pathogenesis [6].

Enteroviruses maintain microbiota balance and are known to promote disease progression and have therefore received considerable attention. Enteroviruses are associated with a variety of diseases such as type 1 and type 2 diabetes, obesity, hypertension, metabolic syndrome, and enteroviral hemorrhagic conjunctivitis [7–9]. Additionally, enteroviruses are associated with CHD and viral myocarditis [10, 11]. Therefore, we aimed to determine the relationship between enteroviruses and CHD by conducting a preliminary study in mice. A growing number of viral metagenomic [12] studies, including metagenomic next-generation sequencing, are being conducted using cost-effective methods [13, 14]. Using viral metagenomic approaches, we collected, processed, sequenced, and performed bioinformatic analysis of gut microbes to gain a more comprehensive understanding of how viral composition and function varies between disease and healthy physiological states of the host [15]. The findings of our study are expected to provide novel insights into the possible roles of enteroviruses in CHD.

## Methods

### Animals

In this study, 15 male adult C57BL/6J mice, aged 42–48 days were used as the sham group, and 15 ApoE^−/−^ mice as the CHD model group. The mice, with a weight of (20 ± 1) g, were purchased from Beijing Weitonglihua Experimental Animal Technology Company. Animal licence number: SCXK-(Beijing) 2016-0006. The mice were fed in the Laboratory animal room of the Institute of Basic Theories of Traditional Chinese Medicine, China Academy of Chinese Medical Sciences. Sterilised water and special or ordinary maintenance feed were freely available and ingested by the animals. The feeding environment was 22 ± 1℃ and the humidity was 55% ± 5%.

### Feeding and model building methods

In the sham group (Sham), mice were fed a conventional chow diet, whereas in the CHD group (CHD), mice were fed a customised high-fat feed (formula containing 78.8% base feed, 10% lard, 10% egg yolk powder, 1% cholesterol, 0.2% sodium cholate). Both feeds were purchased from Beijing Keao Xieli Feed Co., Ltd. Both groups were fed for 12 weeks. At the end of week 8, ligation of the left anterior descending coronary artery was performed to create a CHD mouse model (of a high fat diet combined with ligation of the left anterior descending coronary artery) [16], In the Sham, mice were performed surgery without ligation of the left anterior.

### Cardiac function evaluation

After 12 weeks of feeding, the cardiac function was evaluated using a high-resolution small-animal ultrasound imaging system (FUJIFILM Visualsonics Vevo3100). Ultrasound in M mode was used to record the short axis, and analysis was performed using the ultrasonic imaging system software (Vevo LAB 5.7.0). Data processing and statistical measurement of the left ventricular ejection fraction (EF), left ventricular fractional shortening (FS), stroke volume (SV), cardiac output (CO), Left ventricular anterior wall systolic thickness (LVAWs), Left ventricular anterior wall diastolic thickness (LVAWd), Left ventricular posterior wall systolic thickness (LVPWs), Left ventricular posterior wall diastolic thickness (LVPWd) were evaluated.

### Serum lipid detection

Following anaesthesia, 2,2,2-tribromoethanol (Lot: MKCM646, Merck KGaA) [17] was used to obtain serum from mice. The obtained serum was used to detect total cholesterol (TC), high-density lipoprotein cholesterol (HDL-C), low-density lipoprotein cholesterol (LDL-C), and triglycerides (TG). Total cholesterol (TC; Lot:220,851), high-density lipoprotein cholesterol (HDL-C; Lot:221,691), low-density lipoprotein cholesterol (LDL-C; Lot:221,741), and triglyceride (TG; Lot:220,841) test kits were purchased from Biosino Bio, China.

### Aorta oil red O staining experiment

The chest was opened and the right atrial appendage was cut off; an appropriate amount of sodium chloride was injected from the apex of the heart to perfuse the heart and arterial vessels, and the organs and tissues outside the heart and aorta were cut off. The aorta along the aortic arch to the root of the abdominal aorta was removed under a stereomicroscope, and transferred to a prepared oil red O dyeing solution (Lot: 20,221,212, Beijing Solarbio Science & Technology Co.,Ltd.) for 1 h. Following incubation, the aorta as washed with 60% isopropyl alcohol and ultrapure water to remove excess dyeing solution. The fat outside the lumen was removed and photographed.

### DNA extraction and sequencing

Faecal samples were collected, ground, and nucleic acids were extracted using the CTAB method. Briefly, preheated CTAB extract, 2% mercaptoethanol, and 10% lysozyme was added to the ground tissue sample (ground by swirling centrifugation). After several rounds of centrifugation, the supernatant was obtained and DNA was precipitated using a 75% ethanol solution. The DNA precipitation was dissolved with ultra-pure water and stored in the refrigerator at −20℃ for later use.

Nucleic acid concentration was determined using the Qubit kit (Invitrogen, Qubit3.0, reagent Qubit dsDNA HS Assay kit) according to the manufacturer’s instructions. The integrity was detected by 1% agarose gel electrophoresis. The concentration was ≥ 1 ng/ul, the total amount was greater than or equal to 0.03ug, the main integrity dispersion band was above 5 kb, and there were no obvious impurities below, indicating that the nucleic acid status was normal. Qualified samples were used for library construction using the VAHTS Universal Plus DNA Library Prep Kit for III Iumina ND617. Sequencing was performed using an Illumina NovaSeq 6000 sequencing platform (sequencing strategy: PE150).

### Bioinformatics analysis of virome

Trimmomatic software (V0.33, parameters PELEADING:3 TRAILING:3 SLIDINGWINDOW:50:20 MINLEN:120) was used to remove low-quality raw data and obtain high-quality data. MEGAHIT [18] (default parameters of V1.1.2) was used for metagenomic assembly, to filter contig sequences shorter than 300 bp. The assembly results were evaluated using the QUAST software (default parameters of version V2.3). Matches that covered less than 80% of the sequence length were filtered out and the host genome sequence was eliminated.

Using MMseqs2 software (V12-113e3) to remove redundancy, similarity threshold set to 95%, coverage threshold set to 90%. For specific annotation, the most similar sequences in the Nr database were found by BLAST comparison of the protein sequences of non-redundant genes with the Nr database (diamond V0.9.29.130 comparison screening threshold E-value 1^e − 5^). The annotation information corresponding to the sequence is the annotation information of the gene corresponding to the sequencing genome.

### Non-targeted metabolomics detection of serum metabolites

A UPLC–Q-TOF–MS/MS system (UPLC, ExionLC AD; MS, QTRAP 6500 + System) was used to analyse the sample extracts. The analytical conditions were as follows: UPLC column, Waters Acquity UPLC HSS T3 (1.8 μm 2.1*100 mm); Solvent system, containing 0.1% formic acid-aqueous solution (A) and 0.1% formic acid-acetonitrile solution (B); the gradient started with 2% B (0 min), increased to 98% B (0–10 min), 98% B (10–13 min), and decreases to 2% B (13.1–15 min); Flow rate: 0.4 ml/min; Injection volume: 1 µl. The AB 6500 + QTRAP LC–MS/MS system, equipped with an ESI Turbo ion spray interface, operated in positive and negative ion modes and was controlled by Analyst 1.6 software. ESI source parameters are as follows: Capillary voltage: 2000 V (positive ion mode) or -1500 V (negative ion mode); Cone hole voltage: 30 V; Ion source temperature: 150℃; Desolvent temperature 500℃; Air flow rate: 50 L/h; Desolvent gas flow rate: 800 L/h.

### Statistical analysis

All statistical analyses was performed using SPSS V26.0 and GraphPad Prism V9.0.0 was used to generate graphs .

All metagenomic and metabolomic data analyses were performed using BMK Cloud (www.biocloud.net). Alpha diversity analysis was performed using R V3.1.1 (Picante, V1.8.2) and beta diversity analysis was performed using Python2 (Cogent V1.5.3). Principal coordinate analysis (PCoA) with Binary_Jaccard distance was used to analyse beta diversity differences between the two virus groups, using metagenomeSeq R V3.1.1. Similarity analysis was performed to determine the statistical significance of clustering patterns in the ranking plots. Correlations between the relative abundance of differentially expressed viruses and between differentially expressed viruses and metabolites were determined using Pearson’s correlation analysis.

### Ethical approval

The animal study was reviewed and approved by the Experimental Animal Ethics Committee of the Medical Experimental Research Center, China Academy of Chinese Medical Sciences, approval number: ERCCACMS21-2106-11.

## Results

**Fig. 1:**
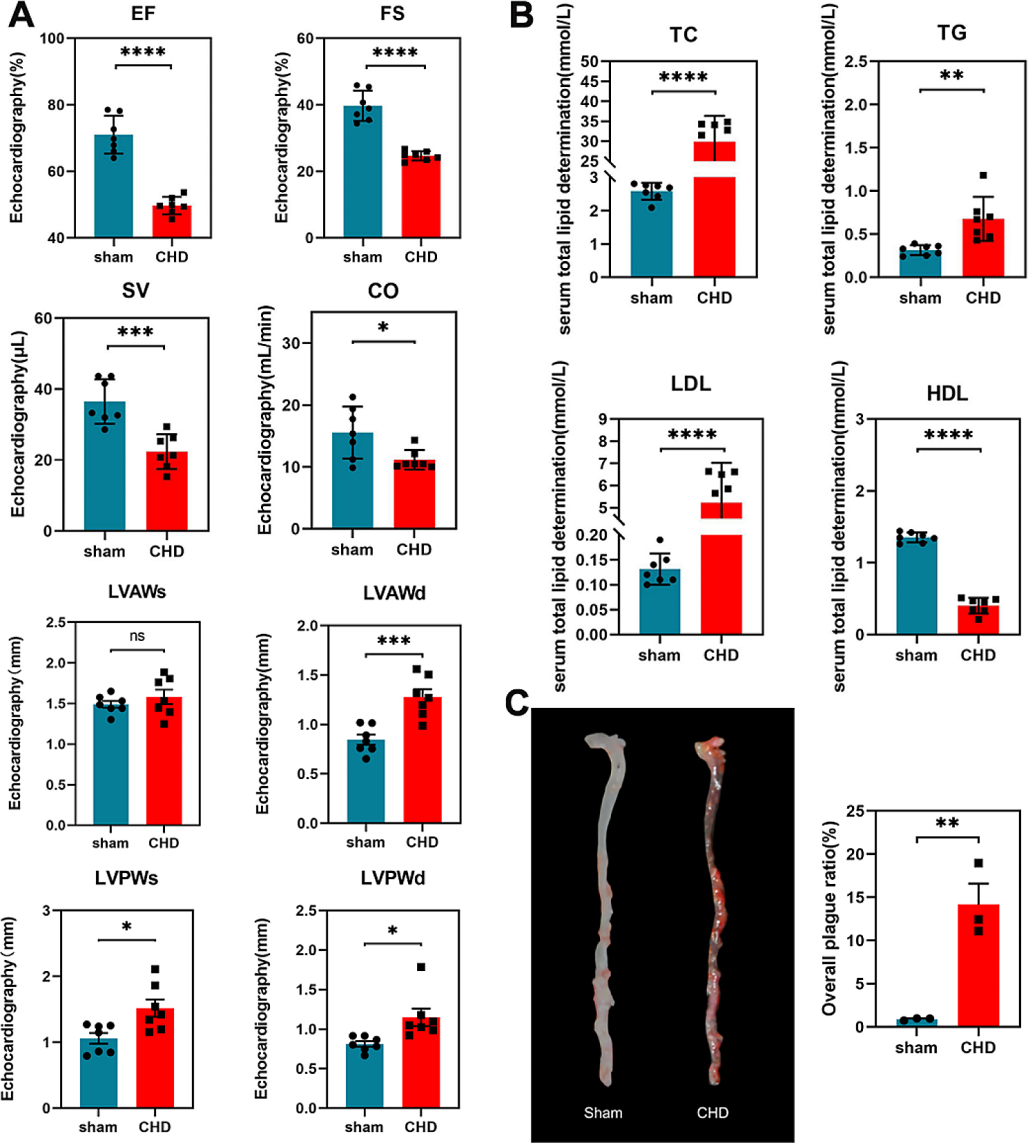
The results of left ventricular function, blood lipid changes, and aorta oil red O staining in mice during the experiment. (**A**) B-mode ultrasound showed changes in cardiac function indexes(*n* = 7). (**B**) Changes of lipid function indexes(*n* = 7). The data are represented as the mean ± standard errors of the means (SEM).*p<0.05, **p<0.01, ***p<0.001, ****p<0.0001.(**C**) Aorta oil red O staining (*n* = 3)

CHD mice model was constructed by a combination of a high-fat diet with ligation of the left anterior descending coronary artery. Changes in cardiac function in CHD mice were evaluated using cardiac ultrasound (*n* = 7), lipid content (*n* = 7), and aortic oil red O staining (*n* = 3). The common indexes EF, FS, SV, CO, LVAWs, LVAWd, LVPWs and LVPWd that reflect left ventricular function (Fig. 1A), and the indexes TC, TG, HDL-C, and LDL-C, that reflect blood lipid levels (Fig. 1B), almost all significantly different between the two mouse groups (CHD model and sham groups). Using aorta oil red O staining, we observed scattered orange-red area staining in the inner wall of the aortic vessels in the CHD model group (Fig. 1C), especially in the branches of the aortic arch. The vessels were relatively dark in colour, These results suggest that we have successfully constructed a model.

**Fig. 2:**
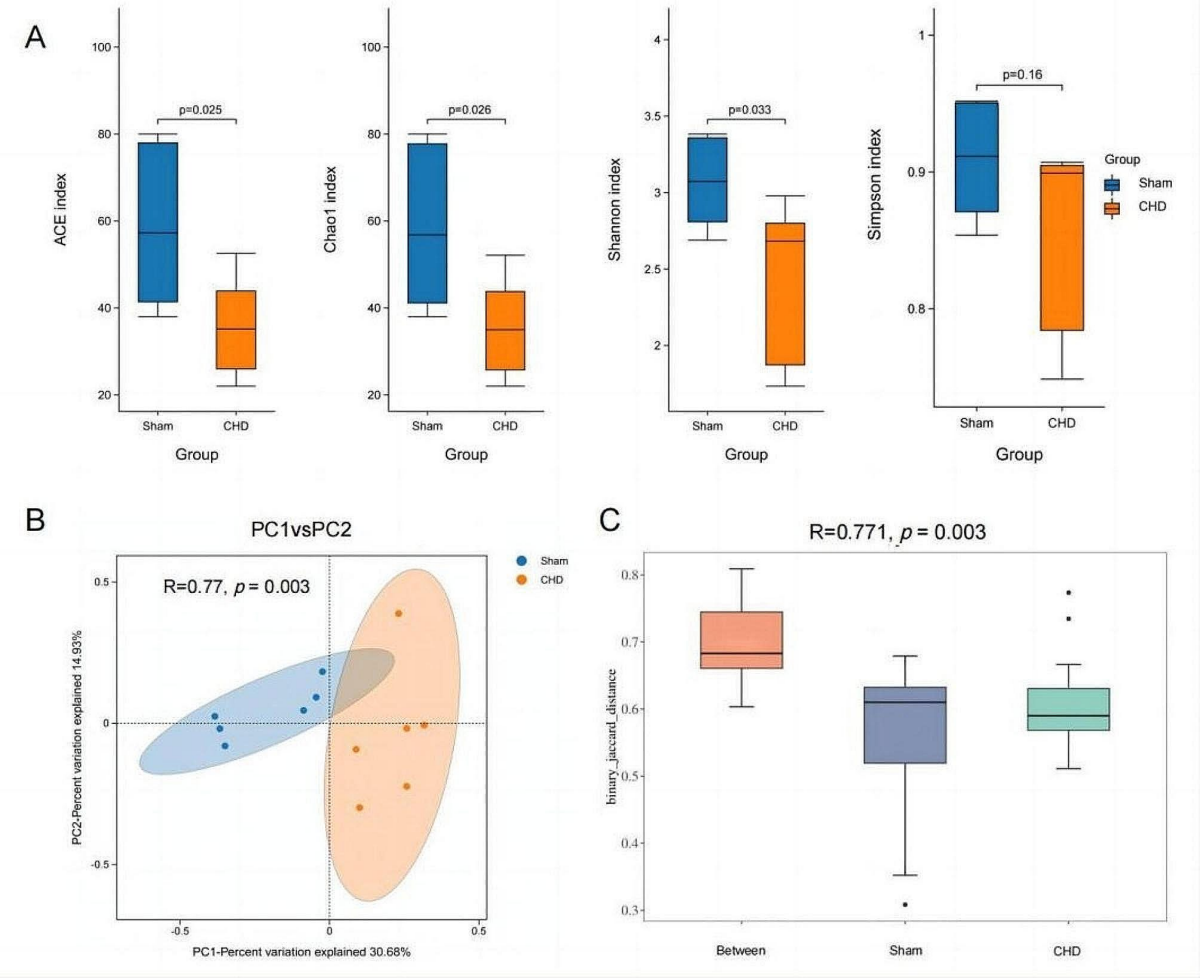
Comparison of viral community diversity between the two groups (sham and coronary heart disease (CHD) groups) (*n* = 6). (**A**) Alpha-diversity determined by ACE, Shannon, Simpson, and Chao1 indices. (**B**) (**C**) Visual representation of Beta-diversity using PCoA plots with Bray-Curtis dissimilar distances. “Between” represents the Beta distance between two groups of samples. The actual range of R values is (− 1, 1), but it is generally between (0, 1). The larger the R, the greater the difference, and the smaller the R, the smaller the difference

Diversity and composition of the virome between the sham and CHD groups were determined. The viral groups in sham and CHD mice faeces were analysed (*n* = 6). Alpha diversity analysis showed significant differences in enterovirus composition between the two groups (ACE index, *p* = 0.025; Chao1 Index, *p* = 0.026; Simpson index, *p* = 0.16; Shannon index, *p* = 0.033; Fig. 2A). Simultaneously, beta diversity was analysed to measure intergroup diversity. PCoA and Binary_Jaccard distance was significantly different between the two groups (the ANOSIM method was used to test the difference between the groups, *R* = 0.771, *p* = 0.003; Fig. 2B).

### Overview of differences between two groups of viruses

**Fig. 3:**
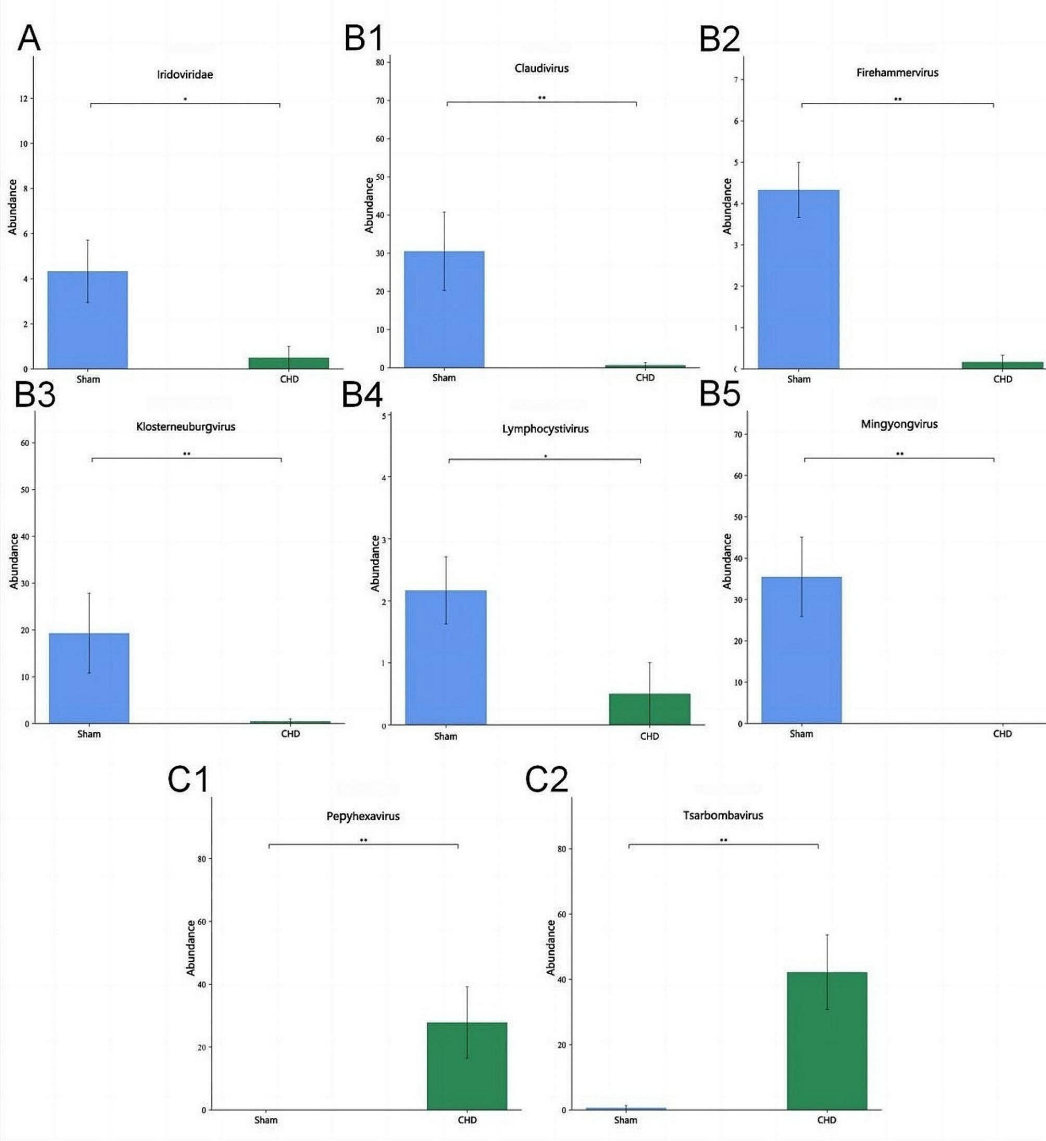
Bar plot of differential viral contigs, at the family and genus levels, between the two groups (sham and coronary heart disease (CHD) groups). The data is represented as the mean ± standard errors of the means (SEM) (*n* = 6), *p<0.05, **p<0.01. (**A**) The relative abundance of viruses varies at the family level. (**B**) The relative abundance of viruses at the genus level decreased significantly in the CHD group. (**C**) The relative abundance of viruses at the genus level was significantly higher in the CHD group

**Fig. 4:**
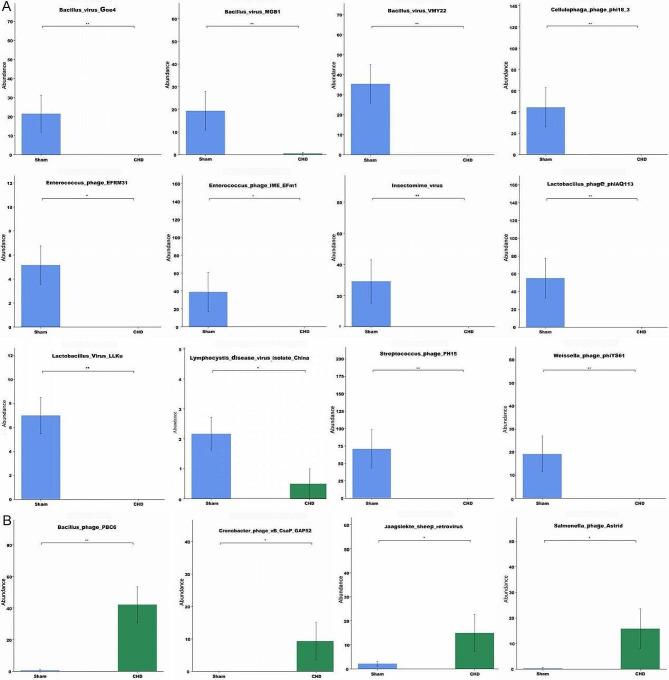
Bar plot of differential viral contigs, species level, between the two groups (sham and coronary heart disease (CHD) groups). The data is represented as the mean ± standard errors of the means (SEM) (*n* = 6), *p<0.05, **p<0.01. (**A**) The relative abundance of different viruses decreased significantly in the CHD group. (**B**) The relative abundance of different viruses significantly increased in the CHD group

We compared the relative abundance of different virions between the two groups, which were successfully annotated at the family, genus, and species levels (*n* = 6). *Iridoviridae*, *Claudiviru*s, *Firehammervirus*, *Klosterneuburgvirus*, *Lymphocystivirus*, and *Mingyongvirus* were enriched in the sham group (Fig. 3AB1-B5), whereas *Pepyhexaviru*s and *Tsarbombavirus* were enriched in the CHD group (Fig. 3C1-C2). In addition, there was a significant difference in the species between the two groups. Cellulophaga_phage_phi18_3, Weissella_phage_phiYS61, Lactobacillus_virus_LLKu, Streptococcus_phage_PH15, Bacillus_virus_MGB1, Insectomime_virus, Lymphocystis_disease_virus_isolate_China, Bacillus_virus_Goe4, Lactobacillus_phage_phiAQ113, Enterococcus_phage_IME_EFm1, Bacillus_virus_VMY22 and Enterococcus_phage_EFRM31 were enriched in Sham group (Fig. 4A). Bacillus_phage_PBC6, Salmonella_phage_Astrid, Jaagsiekte_sheep_retroviru and Cronobacter_phage_vB_CsaP_GAP52 were enriched in CHD group (Fig. 4B).

### Serum metabolic characteristics between the sham and CHD groups

**Fig. 5:**
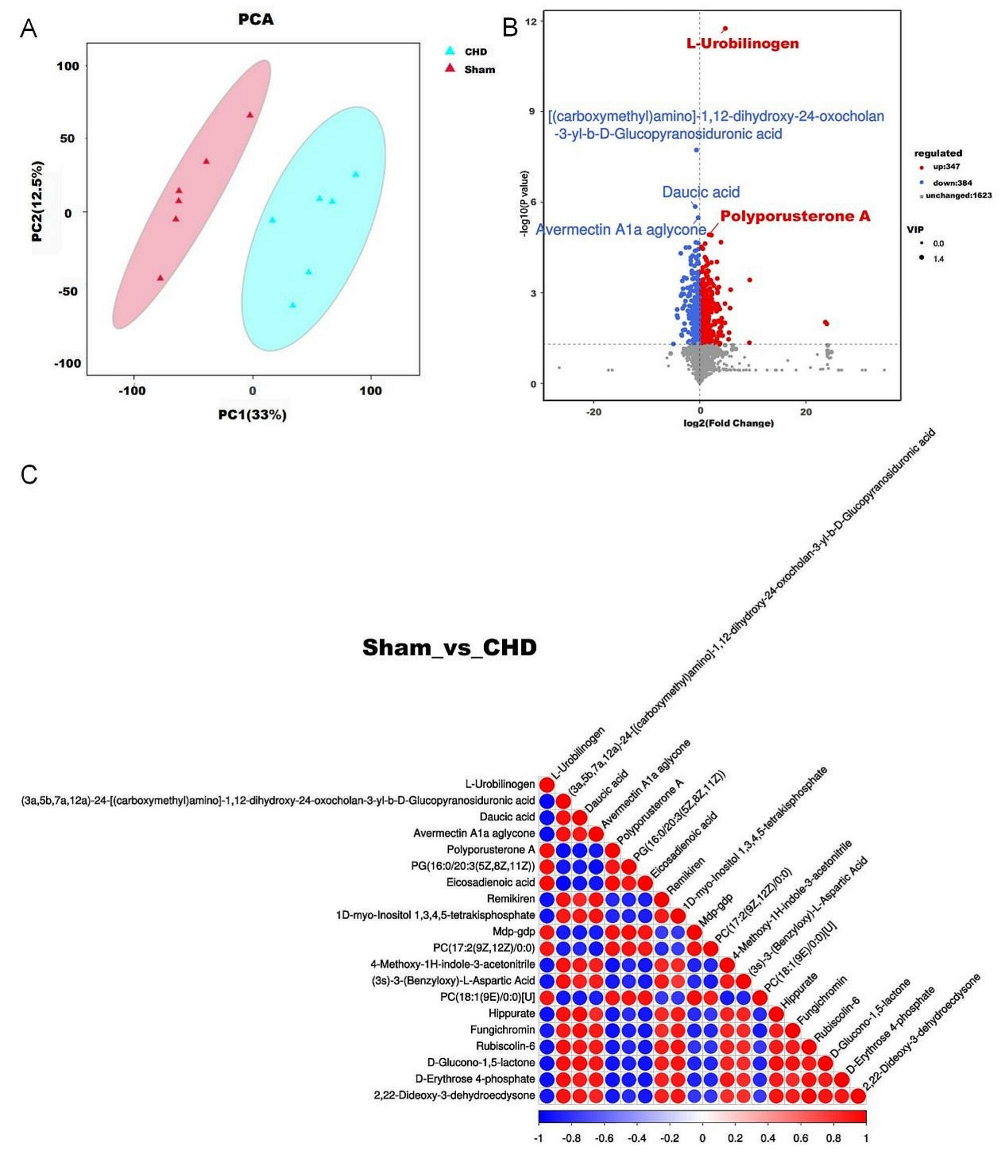
Serum metabolic characteristics of coronary heart disease (CHD) (*n* = 6). (**A**) PCA analysis diagram between groups (sham and CHD groups). (**B**) Volcanic maps of differential metabolites in both groups. (**C**) Correlation diagram of differential metabolites between the groups

UPLC–MS/MS was used to evaluate the metabolic characteristics of the serum samples from both groups (*n* = 6). In this study, 2354 metabolites were identified, and 731 metabolites with variable importance in projection and *p* < 0.05 were identified as differential metabolites. Principal component analysis (PCA) was used to reduce dimensionality and identify specific metabolic characteristics that drive group separation. The PCA score plot revealed significant differences between the two groups (Fig. 5A). A volcanic map with multiple change (FC) > 1.5 and *p* < 0.05 was applied to identify specific metabolite differences between the two groups (Fig. 5B). Some of the metabolites had synergistic or mutually exclusive relationships, and more representative metabolites are shown in the correlation diagram (Fig. 5C).

**Fig. 6:**
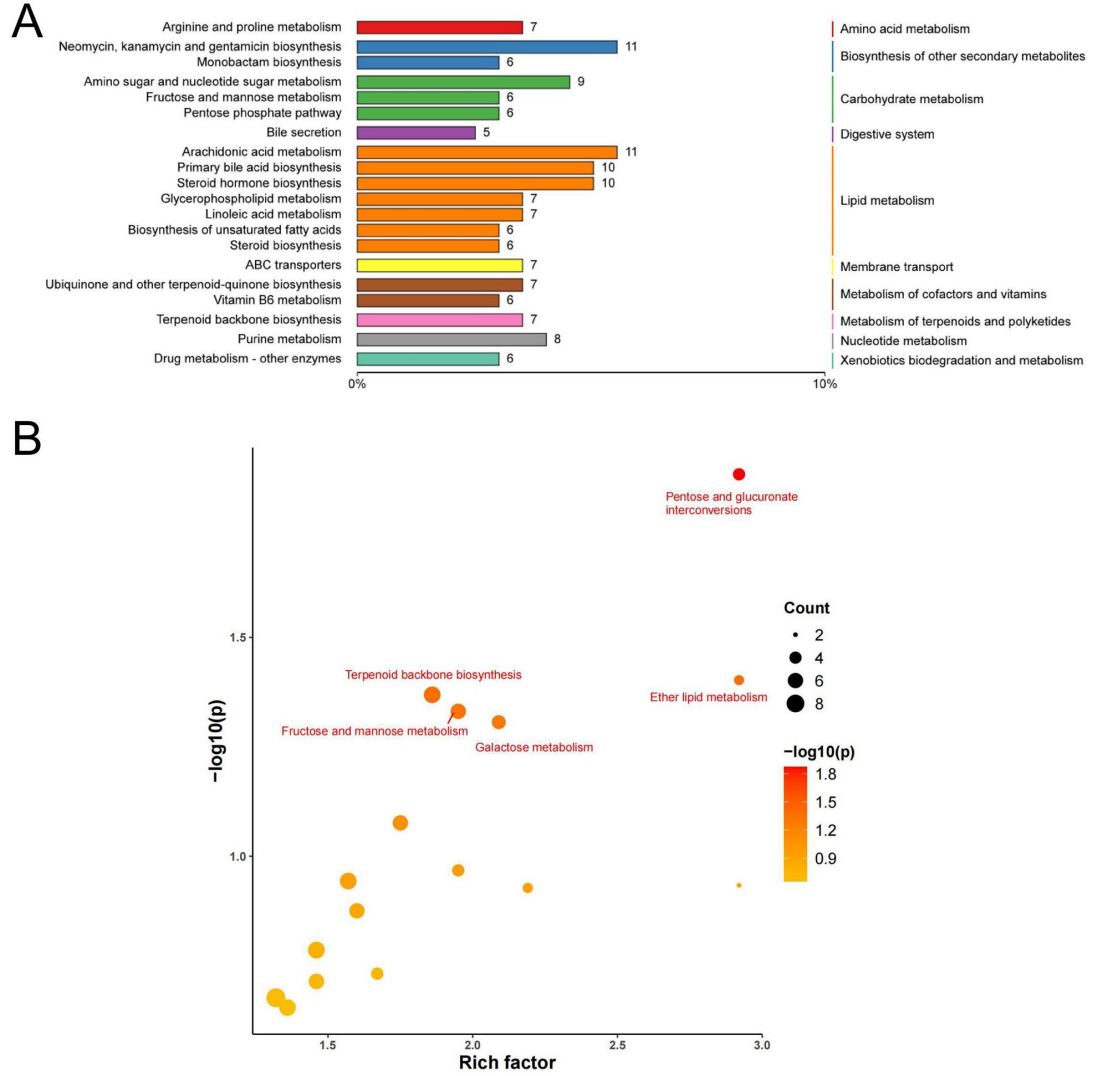
Annotation and enrichment analysis of differential metabolites in coronary heart disease (CHD)(*n* = 6). (**A**) Total bar diagram of differential metabolite metabolic pathways in sham and CHD groups. (**B**) Differential metabolite enrichment analysis of sham and CHD groups

Additionally, the hypergeometric test method of Cluster Profiler was used to enrich the annotation results of the differential metabolite KEGG (Fig. 6A). Most primary metabolic pathways were related to lipid and carbohydrate metabolism. Four differential metabolites were mainly involved in pentose and glucuronate interconversions, namely UDP-glucose, dihydroxyacetone phosphate, l-xylulose 1-phosphate, and UDP-glucuronate; seven in terpenoid backbone biosynthesis, (R)-mevalonate 3-phosphate, aldehyde 3-phosphate, D-aldehyde 3-phosphate, isopentenyl phosphate, 2-C-Methyl-D-erythritol 4-phosphate, isoprene, phytyl diphosphate; and six fructose and mannose metabolism, These are L-Sorbose, L-Fucose 1-phosphate, glyceraldehyde 3-phosphate, dihydroxyacetone phosphate, 2-O-(alpha-D-mannosyl) -d-glycerate, and D-glycerate 3-phosphate, which are mainly involved in galactose metabolism. UDP-glucose, D-Fructose 6-phosphate, aldehyde 3-phosphate, dihydroxyacetone phosphate, d-aldehyde 3-phosphate, glycerophosphocholine, sn-Glycero-3-phosphocholine, and sn-Glycero-3-phosphoethanolmine were involved in ether lipid metabolism (Fig. 6B).

### Co-occurrence network analysis between enteroviruses and metabolites in CHD

**Fig. 7:**
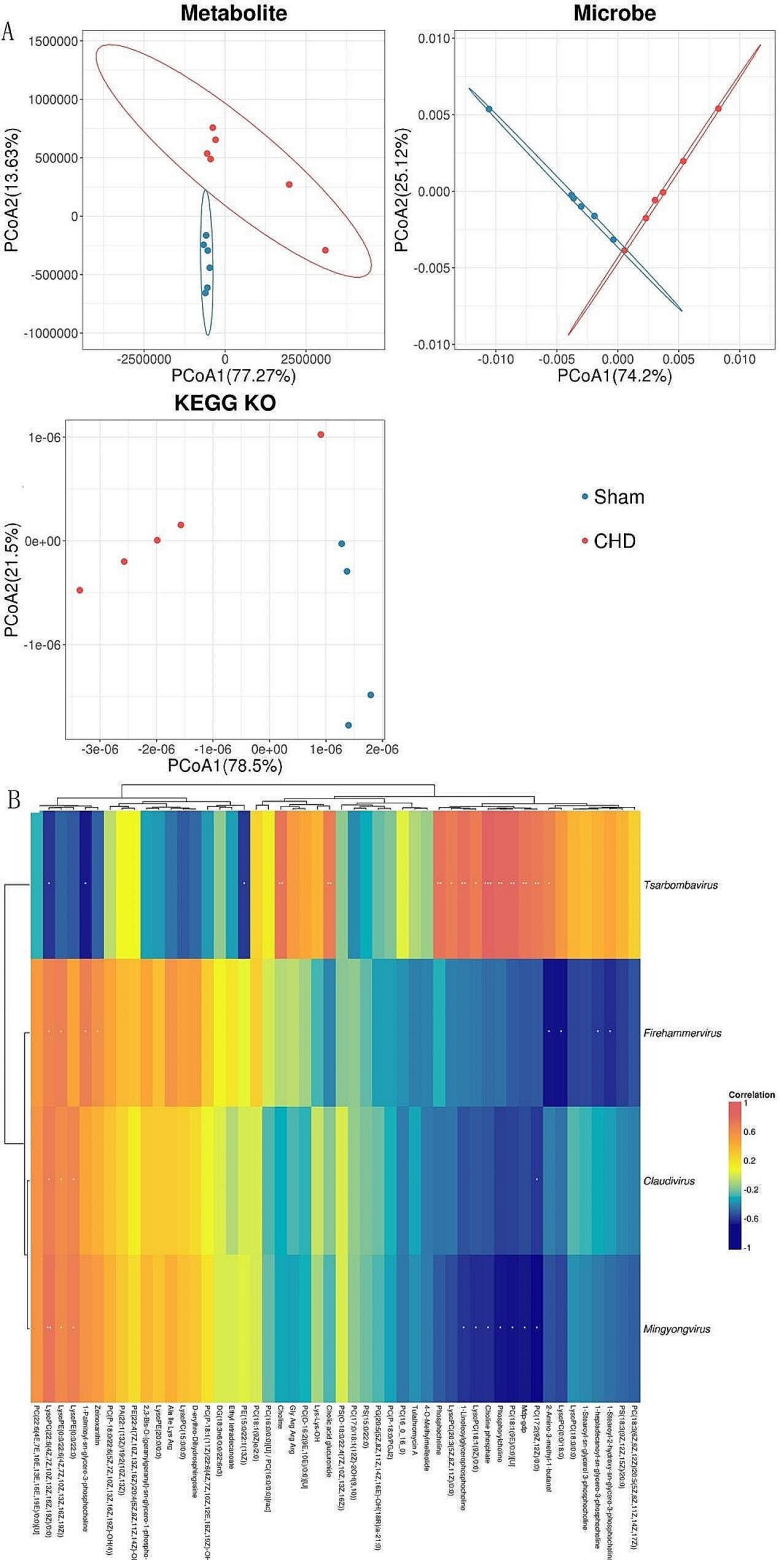
Analysis of the enterovirus group and metabolite co-occurrence network (*n* = 6). (**A**) PCoA analysis (|CC|>0.8, *p* < 0.05). (**B**) Heat map of metabolite clustering and microbial correlation analysis (yellow and orange are positively correlated, and green and purple are negatively correlated)

A co-occurrence network was constructed, based on Pearson correlation analysis, to explore the association between differences in enterovirus groups and metabolites in CHD and sham group. First, PCoA was applied to reflect the differences in multidimensional data on a two-dimensional coordinate chart, and the overall differences in the information of different dimensions between samples were intuitively obtained. The more similar the community compositions of the samples, the shorter the distance between them on the PCoA chart (Fig. 7A).

We identified metabolites such as sn-glycero-3-phosphocholine, glycerophosphocholine, ribostamycin, and eicosadienoic acid, 2, 2,2-dideoxy-3-dehydroecdysone, D-erythrose. 4-phosphate was strongly positively and negatively correlated with viruses in four families: *Tsarbombavirus*, *Firehammervirus*, *Mingyongvirus*, and *Claudivirus*. Moreover, metabolites sn-glycero-3-phosphocholine and glycerophosphocholine were the core differential metabolites of ether lipid metabolism, with significant differences in the differential metabolite enrichment analysis. Both showed strong positive correlations with *Tsarbombaviruses* (Fig. 7B).

## Discussion

The gut microbiome comprises a group of microorganisms that live in the gut of mammalian hosts, including viruses, bacteria, archaea, fungi, and protozoa [19]. It has been established that the gut microbiota is associated with CHD development. However, the underlying mechanisms remain poorly understood. Therefore, we aimed to determine the relationship between enteroviruses and CHD by conducting a preliminary study in mice.

A mouse CHD model, characterised by lipid disturbance and myocardial ischaemia, was established using a high-fat diet combined with ligation of the left anterior descending branch of the coronary artery. To further understand the role of enteroviruses in CHD, the relationship between enteroviruses and serum metabolites was analysed using viral metagenomics and LC–MS/MS methods.

In this study, aortic Oil Red O staining, lipid index detection, and cardiac function detection [20–22] were used. Lipid disturbance and deposition, cardiac function deterioration, and left ventricular structural morphological changes were observed, indicating that a CHD representative animal model was established. Alpha and beta diversity analyses was combined with species composition analysis to assess differences in viral diversity and composition in the data. Between the sham and CHD groups, alpha diversity (Ace, Shannon, Simpson, and Chao1 index) was significantly different, indicating viral diversity between the two groups. The CHD group was less diverse than the sham group; these results are consistent with that of other similar studies [23]. Beta diversity analysis, based on the Binary_Jaccard dissimilarity distance, showed that the sham group could be distinguished from the CHD group. The results showed that the enterovirus group in the CHD mice changed.

Based on intergroup differences, we compared the viral abundance characteristics of the two groups at various taxonomic levels to evaluate specific differences in the viral clusters between the two groups. the viral communities with significant abundance differences between the two groups were *Iridoviridae, Mingyongvirus, Claudivirus, Firehammervirus, Pepyhexavirus, Tsarbombavirus, Klosterneuburgvir us, Salmonella_phage_Astrid, Lymphocystivirus, Bacillus_virus_VMY22, Bacillus_virus_Goe4, Lactobacillus_virus_LLKu*, and *Cronobacter_phage_vB_CsaP_GAP52*. A total of 24 virus communities were observed, and all species were in the family level. Among the virus species with these changes, *Iridoviridae* has been shown to encode viral insulin-like peptides to trigger insulin receptors and insulin-like growth factor receptors [24], indicating that these significant changes in viral colonies have related functions, and their specific mechanisms of action may be complex. Therefore, in this study, the serum metabolome was selected for in-depth research.

Nontargeted metabolomics was used to investigate the potential relationship between differential serum metabolites and the enterovirus group, ultimately identifying 731 differential metabolites in CHD. A large majority of the metabolites are involved lipid metabolic pathways, followed by carbohydrate metabolic pathways. The main pathological basis of atherosclerosis is the deposition of lipids in the inner wall of arteries and the formation of fibrolipopathy [25]. In this study, it was found that arachidonic acid metabolism was the most altered metabolite in the lipid metabolic pathway, and thromboxane A2 is an arachidonic acid metabolite and an effective vasoconstrictor for platelet aggregation and release, which is involved in atherosclerosis [26]. Similarly, the related primary bile acid metabolism is involved in the direct emulsification and solubilisation of lipids, especially in some lipid metabolic diseases, which can enhance the clearance of lipids by enhancing the emulsification and solubilisation of fats, clearing cholesterol, reducing lipid deposition and inflammatory reactions, and increasing the clearance rate of triglycerides [27]. In addition, it shows that lipid metabolism plays an important role in the occurrence and development of CHD. Based on the results, such as the detection of enteroviruses in atherosclatherous plaques [28], we hypothesised that changes in enterovirus groups would participate in the effects of various metabolites on CHD.

Correlation networks were used to investigate the association between differential metabolites and the enterovirus groups. We identified many relationships between the differentially expressed metabolites and enteroviruses. Of particular importance is the metabolites sn-glycero-3-phosphocholine and glycerophosphocholine are core differential metabolites of ether lipid metabolism. These two metabolites showed strong positive correlations with *Tsarbombavirus* [29], is double-stranded DNA myoviruses that can infect various bacilli. Various microorganisms in the genus Bacillus can cause endocarditis [30] and stress the coronary arteries [31]. As a bacteriophage virus, it is conceivable that tsarbombavirus affects the Bacillus family and heart health. Given its importance, we believe that it is a promising EV species. *Firehammervirus* is a genus of *Campylobacter phages* [32]. However, little research has been conducted on this genus to determine its relationship to cardiovascular health. *Mingyongvirus* and *Claudivirus* are similar to *Firehammervirus*.

In addition, correlation analysis showed that other significantly related metabolites were important in linoleic acid metabolism, unsaturated fatty acid biosynthesis, glycerol phospholipid lipid metabolism, choline metabolism, and other pathways, with the first three metabolites belonging to the secondary metabolic pathways of lipid metabolism. However, it has been confirmed that there is a clear causal relationship between phages and metabolites; that is, phages can regulate neurotransmitter metabolites and the mammalian host effects of metabolites. These metabolites are involved in amino acid and bile acid metabolism [33].

The viral communities associated with these metabolites include *Tsarbombavirus*, *Firehammervirus*, *Mingyongvirus*, and *Claudivirus*. The relationship among these viral communities, their metabolites, and CHD has not yet been elucidated. However, based on the literature and functional localisation of phages, we strongly believe that viral communities can affect the metabolites associated with CHD. The *Tsarbombavirus* identified in this study may be a promising bacteriophage to regulate the role of microbiota and metabolism associated with CHD. The next step is to carry out in-depth research on these promising bacteriophages, and propose more effective and targeted prebiotics, probiotics, antibiotic therapies, etc.

## Conclusions

In conclusion, this study showed that the enterovirus, *Tsarbombavirus*, changes with ether lipid metabolism. Viruses may influence the pathophysiology of CHD through interactions with other microorganisms, and results in changes in body metabolism and influence the course of the disease, as well as three groups of enterovirus components related to lipid metabolism.

## Data Availability

The data presented in the study are deposited in the NCBI repository, accession number PRJNA1012496.

## Abbreviations

CO: Cardiac output
CHD: Coronary heart disease
CRISPR: Clustered regularly interspaced short palindromic repeats
CRT: CRISPR recognition tool
HDL-C: High-density lipoprotein cholesterol
EF: Left ventricular ejection fraction
FS: Left ventricular fractional shortening
LDL-C: Low-density lipoprotein cholesterol
PCA: Principal component analysis
PCoA: Principal coordinate analysis
SEM: Standard error of the mean
SV: Stroke volume
LVAWs: Left ventricular anterior wall systolic thickness
LVAWd: Left ventricular anterior wall diastolic thickness
LVPWs: Left ventricular posterior wall systolic thickness
LVPWd: Left ventricular posterior wall diastolic thickness
TC: Total cholesterol
TG: Triglycerides
UPLC-Q-TOF-MS/MS: Ultra-performance liquid chromatography–quadrupole time-of-flight–tandem mass spectrometry
